# Identification of Hub Genes as Biomarkers Correlated with the Proliferation and Prognosis in Lung Cancer: A Weighted Gene Co-Expression Network Analysis

**DOI:** 10.1155/2020/3416807

**Published:** 2020-06-10

**Authors:** Xuting Xu, Limin Xu, Huilian Huang, Jing Li, Shunli Dong, Lili Jin, Zhihong Ma, Liqin Li

**Affiliations:** Huzhou Key Laboratory of Molecular Medicine, Huzhou Cent Hospital, Affiliated Cent Hospital HuZhou University, Huzhou, Zhejiang 313000, China

## Abstract

Lung cancer is one of the most malignant tumors in the world. Early diagnosis and treatment of lung cancer are vitally important to reduce the mortality of lung cancer patients. In the present study, we attempt to identify the candidate biomarkers for lung cancer by weighted gene co-expression network analysis (WGCNA). Gene expression profile of GSE30219 was downloaded from the gene expression omnibus (GEO) database. The differentially expressed genes (DEGs) were analyzed by the limma package, and the co-expression modules of genes were built by WGCNA. UALCAN was used to analyze the relative expression of normal group and tumor subgroups based on tumor individual cancer stages. Survival analysis for the hub genes was performed by Kaplan–Meier plotter analysis with the TCGA database. A total of 2176 genes (745 upregulated and 1431 downregulated genes) were obtained from the GSE30219 database. Seven gene co-expression modules were conducted by WGCNA and the blue module might be inferred as the most crucial module in the pathogenesis of lung cancer. In the pathway enrichment analysis of KEGG, the candidate genes were enriched in the “DNA replication,” “Cell cycle,” and “P53 signaling pathway” pathways. Among these, the cell cycle pathway was the most significant pathway in the blue module with four hub genes CCNB1, CCNE2, MCM7, and PCNA which were selected in our study. Kaplan–Meier plotter analysis indicated that the high expressions of four hub genes were correlated with a worse overall survival (OS) and advanced tumors. qRT-PCR showed that mRNA expression levels of MCM7 (*p* = 0.038) and CCNE2 (0.003) were significantly higher in patients with the TNM stage. In summary, the high expression of the MCM7 and CCNE2 were significantly related with advanced tumors and worse OS in lung cancer. Thus, the MCM7 and CCNE2 genes can be good indicators for cellular proliferation and prognosis in lung cancer.

## 1. Introduction

Lung cancer is one of the most common malignant tumors in the world [1]. According to the data released by the World Health Organization in 2019, the incidence and mortality of lung cancer in all kinds of malignant tumors in the world are the highest [2]. The number of deaths from lung cancer exceeds the sum of the deaths of three malignant tumors of breast cancer, prostate cancer, and colorectal cancer. Obviously, lung cancer has become a widespread and serious public health problem worldwide.

Incidence of lung cancer is insidious, and the pathogenesis is complex; it is easy to be ignored by patients and miss the best treatment opportunity. More than 85% of patients have been in advanced stage when clinical diagnosis and lost the opportunity of surgery; the survival rate of lung cancer is very low which is reported to be closely related to the tumor stage. After effective treatment, the 10-year survival rate of patients with stage IA can reach 92%, while the five-year survival rate of patients with stage IV is only 4.2%, which shows the importance of early diagnosis and treatment to reduce the mortality of patients with lung cancer [3].

The development of tumor biomarkers is one of the means of early diagnosis of lung cancer, but only from a local focus on a single or a certain gene cannot meet the regulation of this highly complex tumor. Based on the whole regulation network, some genes in tumors are abnormally expressed and closely related to many other genes. Their expression may play an important role in the occurrence and development of tumors. Such genes in the regulatory network hub become hub genes. In recent years, with the rise of high-throughput sequencing technologies such as gene chips and RNA-seq, large-scale omics data has been generated. Further data analysis and mining will facilitate a systematic study of the association of multiple gene expressions. Weighted correlation network analysis (WGCNA) is a systematic biological method used to describe the pattern of gene association between different samples [4]. It can be used to identify highly synergistically altered gene sets based on the intrinsicality of gene sets. Association with gene sets and phenotypes identifies candidate biomarker genes or therapeutic targets. Compared to genes that only focus on differential expression, WGCNA uses thousands or nearly 10,000 of the most variable genes or all of the genes to identify the set of genes of interest, to make a significant association analysis with a given phenotype. Therefore, the results obtained by this method have more biological significance and higher reliability [5, 6].

In the present study, the WGCNA method was applied to analyze the gene expression dataset to identify the candidate biomarkers for lung cancer based on the TNM stage of lung cancer patients.

## 2. Materials and Methods

### 2.1. Data Sources and Data Preprocessing

Gene expression profile of GSE30219 [7] was downloaded from the gene expression omnibus (GEO) database of the National Center for Biotechnology Research (http://www.ncbi.nlm.nih.gov/gds). The GSE30219 data contained 307 lung tissue samples (including 14 normal tissues and 293 case tissues) analyzed with the Affymetrix Human Genome U133 Plus 2.0 Array. Clinical characteristics were downloaded at the same time. All the data processing and analysis were carried out by the R programming language (v3.6.1).

### 2.2. Differentially Expressed Genes (DEGs) Screening and Enrichment Analysis

We used the limma package [8] to perform normalization and log2 conversion for GSE30219 data. The threshold for identifying DEGs was set at a ∣log2 (fold change) | >1.2 and adjust *p* value <0.05. The functional enrichment analysis for DEGs was including Gene Ontology (GO) and Kyoto Encyclopedia of Genes and Genomes (KEGG: http://http://www.kegg.jp/kegg/) pathway enrichment analysis, which was performed for genes by clusterProfiler package in R [9]. An adjusted *p* value smaller than 0.05 is considered significant.

### 2.3. Clinically Significant Module and Hub Gene Identification

Upregulated and downregulated DEGs were selected for weighted gene co-expression network analysis (WGCNA) according to standard WGCNA R package procedures [10, 11]. To obtain a high-scale independence and average connectivity, the soft-thresholding power value was calculated from a gradient test range from 1 to 20 and determined by pickSoftThreshold function. Then, we calculated the topological overlap matrix (TOM) according to the corresponding soft-thresholding power.

The module eigengene (ME) was calculated to evaluate correlations between the modules and different clinical traits. The biologically significant modules were evaluated in linear regression between gene significance (GS) and clinical traits by Pearson's correlation test. The modules with the higher correlation were identified in the further analysis. We screened the hub genes by the association between gene and module or clinical trait. All the genes met both of the following conditions of cor. module membership (MM) > 0.8 and cor. gene significance > 0.5 which were regarded as candidate hub genes. Protein-protein network (PPI) of these candidate hub genes were analyzed by STRING (https://string-db.org). In our study, we set a combined score of 0.7 as the cutoff value. The KEGG pathway analysis was used to carry out for the candidate hub genes. To identify the key hub genes from the candidate ones, ANOVA was used to estimate the relative expression of the normal sample and subgroup lung cancer sample.

### 2.4. TCGA Data and Survival Analysis of Hub Genes

UALCAN (http://ualcan.path.uab.edu) was an open-access interactive web resource for analyzing cancer OMICS data. It is used to analyze the relative expression of normal group and tumor subgroups based on tumor individual cancer stages. Survival analysis for the hub genes was performed by Kaplan–Meier plotter analysis (http://www.kmplot.com) with the TCGA database. And GEPIA (http://gepia.cancer-pku.cn/index.html) was used to evaluate the expression levels of hub genes. It is an interactive network server for analyzing the sequencing expression of RNA data from the GTEx projects and TCGA.

### 2.5. Subjects and Clinical Data

Fresh tissue specimens were obtained from 59 patients who underwent surgical resection of lung cancer at the Huzhou Central Hospital from 2015 to 2019. All the patients were received without any chemotherapy or radiation treatment prior to the surgery. The tissue samples were immediately frozen in liquid nitrogen and stored at -80°C before RNA isolation. Our study was approved by the ethics committees of the Huzhou Central Hospital. And written informed consent forms were acquired from all of the participants.

### 2.6. RNA Extraction and Quantitative Real-Time PCR (qRT-PCR)

Total RNA was extracted from tissue specimens by using TRIzol™ Reagent (Thermo Fisher Scientific). The isolated RNA was converted to cDNA by the Prime-Script RT reagent kit (TaKaRa, Dalian, China). Quantitative real-time PCR analysis was conducted with the SYBR Premix Ex Taq TM II kit (TaKaRa) on an ABI 7500 Real-Time PCR System (Applied Biosystems, USA) and normalized to the expression of *β*-actin.

### 2.7. Statistics

All the stoical analysis was conducted by the R programming language (version 3.6.0). ROC curve analysis was used to calculate the area under the ROC curve.

### 2.8. Statistical Analysis

Statistics 18.0 software (SPSS Inc., Somers, NY, USA) was used to perform the statistical analyses in the study. *χ*^2^ test was used to determine statistical significance. *p* < 0.05 was considered statistically significant.

## 3. Result

### 3.1. Identification of DEGs and Enrichment Analysis

**Figure 1:**
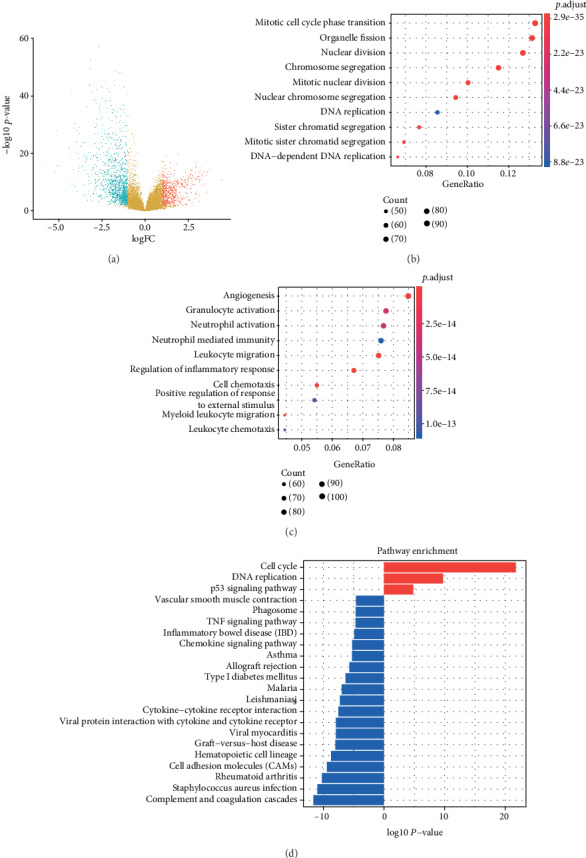
Identification of DEGs and enrichment analysis in GSE30219 between lung cancer and normal tissues: (a) volcano plot of the DEGs; (b, c) GO enrichment analysis of upregulated (b) and downregulated (c) DEGs; (d) KEGG pathway enrichment analysis of upregulated and downregulated DEGs.

All of 293 lung cancer samples and 14 normal samples were analyzed in our study. According to our cutoff criteria (∣log2 (fold change) | >1.2 and adjust *p* value <0.05), 745 upregulated and 1431 downregulated DEGs were identified between the cancer group and the normal one (Figure 1(a)).

After DEGs being obtained, we performed GO and KEGG pathway enrichment analysis to explore the biological functions of these DEGs. The top GO terms in upregulated DEGs were Mitotic cell cycle phase transition, Organelle fission, Nuclear division, Chromosome segregation, and Mitotic nuclear division (Figure 1(b)). As for the downregulated DEGs, the top 5 enriched GO terms were including Angiogenesis, Granulocyte activation, neutrophil activation, neutrophil-mediated immunity, and leukocyte migration (Figure 1(c)). As shown in Figure 1, the upregulated DEGs were enriched in KEGG pathway of cell cycle, DNA replication, and P53 signaling pathway, and downregulated DEGs were mainly associated with Complement and coagulation cascades, Staphylococcus aureus infection, Rheumatoid arthritis, and cell adhesion molecules (Figure 1(d)).

### 3.2. Identification of Co-Expression Gene Modules

**Figure 2:**
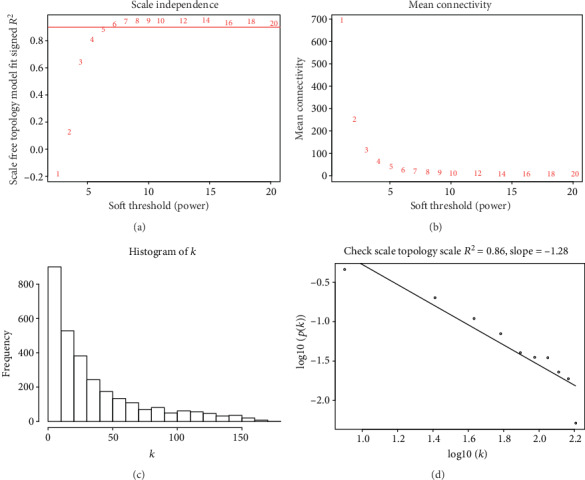
(a) Effects of power values on the scale independence of DEGs co-expression modules; (b) effects of power values on the average connectivity of DEGs co-expression modules; (c) histogram of connectivity distribution when power = 5; (d) check the scale-free topology when power = 5.

After removing the normal samples, we included the 2176 identified DEGs and 293 lung cancer samples to construct co-expression gene network in total. The scale-free fit index and mean connectivity were used to calculate the power of *β* for further analysis. When the power of *β* = 5, the scale-free fit index was over 0.8 (Figure 2, *R*^2^ = 0.86).

**Figure 3:**
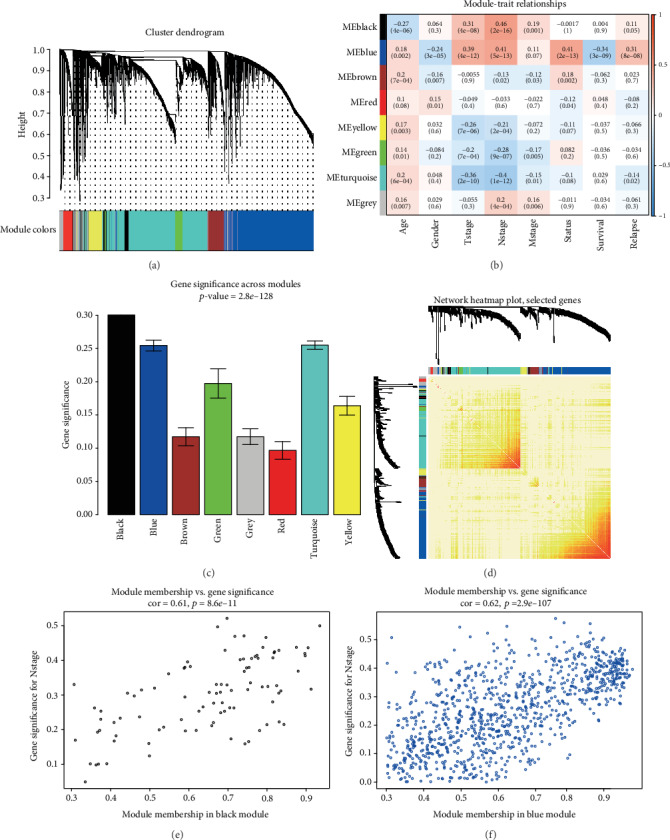
Identification of co-expression gene modules and the association with clinical traits. (a) Clustering dendrogram of DEGs; (b) heatmap of correlation between ME and clinical traits of age, gender, T stage, N stage, M stage, alive status, survival time, and relapse status; (c) distribution of GS in seven modules; (d) the TOM plot of 400 genes; (e, f) scatter plot of ME in the black (e) and blue (f) modules.

Seven co-expression modules were identified via average linkage clustering (Figure 3(a)). Our result indicated that the black module had the highest association with the clinical parameters (Figure 3(b), *R*^2^ = 0.46, *p* = 2*e* − 16). Notably, the blue module had the second-highest association with N stage (Figure 2(b), *R*^2^ = 0.41, *p* = 5*e* − 13) and also had stronger association with T stage (Figure 3(b), *R*^2^ = 0.39, *p* = 4*e* − 12), alive statue (Figure 3(b), *R*^2^ = 0.41, *p* = 2*e* − 13), survival time (Figure 3(b), *R*^2^ = −0.34, *p* = 3*e* − 09), and relapse (Figure 2(b), *R*^2^ = 0.31, *p* = 8*e* − 08). Black and blue modules were the two top modules with closer correlation of the N stage based on the gene significance measure (Figure 3(c)). In addition, it implied the statistically significant correlation between module membership and GS in black (Figure 3(e): cor = 0.61, *p* = 8.6*e* − 11) and blue (Figure 3(f): cor = 0.62, *p* = 2.9*e* − 107) models in the current study.

### 3.3. PPI Network and Validation of Hub Genes

**Figure 4:**
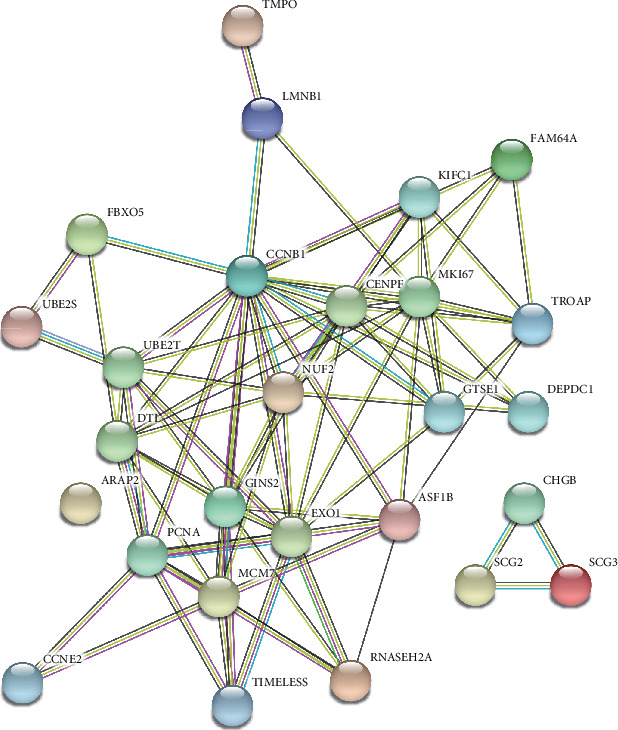
Protein-protein interaction network of hub-genes.

14 genes in the black model and 25 genes in the blue model were carried out by the criteria of cor. MM > 0.8 and cor. GS > 0.5. We mapped the 39 genes to the STRING database and analyzed with a combined score > 0.7. As shown in Figure 4, we found several candidate hub genes. Furthermore, KEGG pathway analysis indicated seven genes, including MCM7, PCNA, RNASEH2A, CCNB1, CCNE2, GTSE1, and FBXO5. The seven genes were enriched in five KEGG pathways (Table 1). We found that the four hub genes (CCNB1, CCNE2, MCM7, and PCNA) enriched in the cell cycle pathway were the most important genes, which played significant roles in other pathways.

**Figure 5:**
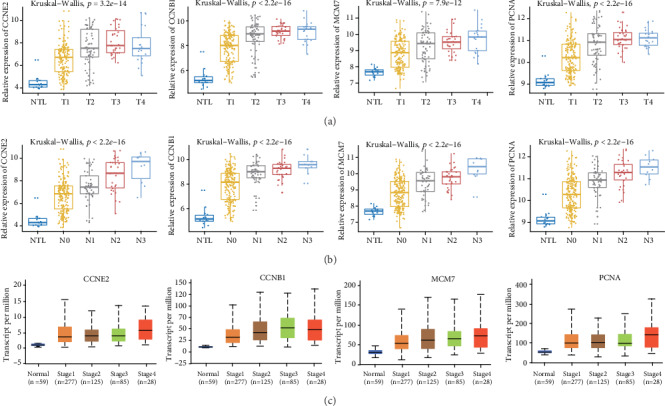
Aberrant expression of the four hub genes can be a candidate biomarker in lung cancer. (a, b) expression levels of four hub genes in GSE30219 were positively correlated with T stage (a) and lymph node metastasis N stage (b); (c) validation of our hub genes was positively correlated with stage by TCGA data.

**Figure 6:**
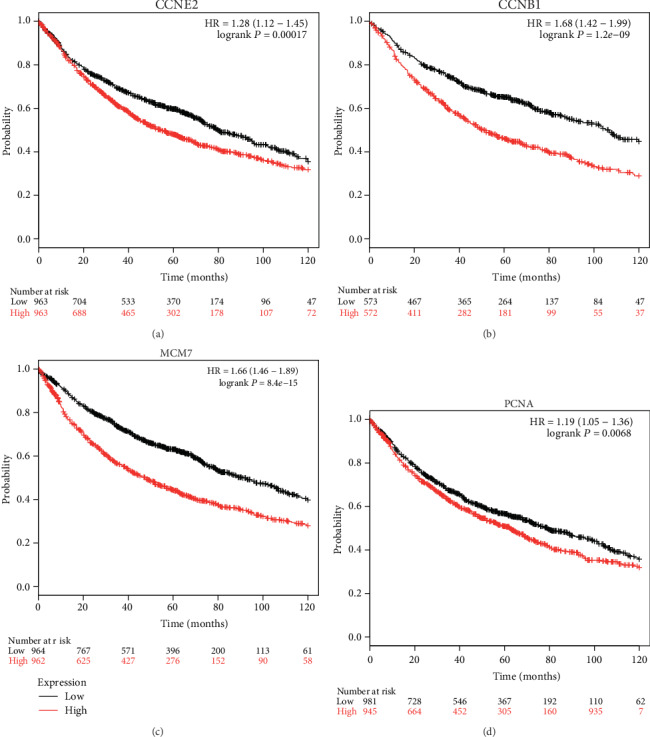
The survival analysis of four hub genes by the KM-plotter. (a) CCNE2; (b) CCNB1; (c) MCM7; (d) PCNA.

Our results found that tumor with larger size (T stage) or with more lymph node metastasis (N stage) had higher mRNA levels of four hub genes from GSE30219 (Figure 5, *p* < 0.05). The further analysis of lung cancer data from the TCGA database showed similar results with GSE30219 (Figure 5, *p* < 0.05). Nevertheless, the four hub gene expressions had a significant relationship with other clinical characteristics, such as relapse and several times. The positive prognostic effect of four hub genes was also supported by Kaplan–Meier plotter analysis. It indicated that higher expressions of CCNE2 (Figure 6(a): HR = 1.28 (1.12−1.45), *p* = 0.00017), CCNB1 (Figure 6(b): HR = 1.68 (1.42−1.99), *p* = 1.2*e* − 09), MCM7 (Figure 6(c): HR = 1.66 (1.46−1.89), *p* = 8.4*e* − 15), and PCNA (Figure 6(d): HR = 1.19 (1.05−1.36), *p* = 0.0068) were correlated with a worse overall survival (OS). These hub genes were highly expressed in tumor samples and had a close relationship with advanced tumors. ROC curve analysis showed that the four hub genes had good diagnostic performance in distinguishing cancer from the normal by using the dataset of GSE30219, AUC was 0.966 for CCNB1, 0.949 for CCNE2, 0.916 for MCM7, and 0.944 for PCNA (Table 2). The results from GEPIA showed that all the four hub genes were higher expression levels in the cancer than the normal one (Supplementary Figure 1). The expression of CCBN1 and MCM7 was showed the statistical significance between the different TNM stages (Supplementary Figure 2). qRT-PCR showed that mRNA expression levels of MCM7 (*p* = 0.038) and CCNE2 (0.003) were significantly higher in patients with the TNM stage (Table 3).

## 4. Discussion

Lung cancer is the most common cancer all around the world, with high morbidity and mortality. Although early screening by computed tomography (CT) reduces the associated mortality [12], tumor invasion and migration-mediated disease progression represent the leading cause of cancer-related death [13].

The identification of disease-associated modules via WGCNA which focused on the relationship between gene co-expression modules has emerged as a powerful and reliable method of obtaining novel insights into cancer biology [10, 14]. WGCNA was widely used in various biological processes analysis to process differently expressed genes and interactions among genes [15–17]. These hub genes can be identified as therapeutic targets or candidate biomarkers in many diseases [5, 18–20]. Combined with the RNA-seq data and WGCNA analysis, Wei et al. found that genes involved in cell adhesion, ECM-receptor interaction, focal adhesion, and PI3K-Akt signaling pathway play crucial roles in human lung adenocarcinomas [15].

In the present study, the gene expression patterns obtained from the GSE30219 database revealed a total of 2176 genes, including 745 upregulated and 1431 downregulated genes, which were differently expressed in lung cancer samples compared with controls. Furthermore, 7 gene co-expression modules were conducted by WGCNA based on 2,176 DEGs from 293 lung cancer samples. Through analyses, the blue module might be inferred as the most crucial module in the pathogenesis of lung cancer. A total of 25 genes with high connectivity in the blue module were distinguished as candidate hub genes. In further pathway enrichment analysis of KEGG, these genes were enriched in the “DNA replication,” “Cell cycle,” and “P53 signaling pathway” pathways. The cell cycle was the most significant pathway in the blue module with four hub genes CCNB1, CCNE2, MCM7, and PCNA which were selected in our study. And we confirmed that the mRNA expression levels of MCM7 (*p* = 0.038) and CCNE2 (0.008) were significantly higher in patients with the TNM stage (Table 3) in patient trusses.

The cell cycle is a complex process that involves numerous regulatory proteins that direct the cell through a specific sequence of events which a cell duplicates its genome, grows, and divides [21, 22]. Deregulation of the cell cycle is a common event in lung cancer. Usually, several defects of cell cycle regulation are concomitant and have a cumulative adverse effect on prognosis [23]. Cell cycle-related genes such as Ki-67, P53, P16, and RB1 were reversed as proliferative and prognosis markers in many diseases [24–27]. A study demonstrated that the Ki-67 proliferation index is a clinically meaningful biomarker in NSCLC that allows reliable estimation of prognosis [28]. The combination of P53/P21 expression and smoking history may be a useful biomarker for tumor progression and prognosis of NSCLC patients [29].

CCNE2 are important members of the cyclin family which function as regulators of the cell cycle by activating cyclin-dependent kinase (CDK) enzymes [30, 31]. They are crucial cell-cycle regulators in the G2/M phase and in G1/S transition separately in cell proliferation and differentiation [32, 33]. A study has demonstrated that CCNE2 may be useful as diagnostic biomarker for early detection of gastric carcinoma for its overexpression in early stages of gastric carcinoma which significantly correlated with differentiation, invasion, and metastasis [34]. In addition, CCNE2 was the target of miR-3607-3p and miR-30d-5p in NSCLC to inhibit tumor cell proliferation and metastasis [35, 36]. MCM proteins including MCM7 have been utilized as diagnostic and prognostic tumor markers for their higher specificity and sensitivity than the conventional proliferative markers, such as Ki-67 and PCNA [37, 38]. A study [39] found that MCM7 expression elevated with gastric tumor grade increasing and had a positive correlation with Ki-67 significantly. The combination of MCM7 and Ki67 may serve as more sensitive proliferation markers for the evaluation of gastric carcinoma [39]. Furthermore, the higher expression of MCM7 was associated with poorer prognosis of the hepatocellular carcinoma patients and NSCLC patients than controls [38, 40].

There were several limitations in our work. Firstly, the normal sample size of GSE30219 was rather small, which might lead to a mistake in finding markers with small effects. More subjects especially normal samples were needed to enhance the statistical power. Secondly, more patients' tissue samples with the diagnosis of TNM were needed to further verify the relationship between the hub genes and TNM stage.

In our research, the higher expression of the four hub-genes was significantly related with advanced tumors and worse OS in lung cancer. Thus, CCNE2 and MCM7 can be good indicators for cellular proliferation and prognosis in lung cancer.

## Figures and Tables

**Table 1:** The ROC curve for prediction of NSCLC based on the expression level of hub genes. KEGG pathway analysis of 39 candidate hub genes mapped to STRING database.

ID	KEGG pathway	Count	FDR	Gene symbol
hsa03030	DNA replication	3	0.003	MCM7, PCNA, RNASEH2A
hsa04110	Cell cycle	4	0.003	CCNB1, CCNE2, MCM7, PCNA
hsa04115	p53 signaling pathway	3	0.0059	CCNB1, CCNE2, GTSE1
hsa03430	Mismatch repair	2	0.0132	EXO1, PCNA
hsa04114	Oocyte meiosis	3	0.016	CCNB1, CCNE2, FBXO5

**Table 2:** Prediction performance of expression level of hub genes between normal and lung cancer.

Gene	AUC (95% IC)	Sensitivity (%)	Specificity (%)
CCNB1	0.966 (0.93-1)	0.925	0.929
CCNE2	0.949 (0.90-0.99)	0.973	0.643
MCM7	0.916 (0.87-0.96)	0.785	1.000
PCNA	0.944 (0.89-0.99)	0.894	0.929

**Table 3:** The association between TNM stage and four hub genes by qPCR.

	CCNE2		*p* value
TNM stage	Low expression	High expression	
I	11	16	0.003
II	15	3	
III/IV	4	10	
	CCNB1		*p* value
TNM stage	Low expression	High expression	
I	15	12	0.067
II	9	8	
III/IV	3	12	
	MCM7		*p* value
TNM stage	Low expression	High expression	
I	15	12	0.038
II	10	7	
III/IV	3	12	
	PCNA		*p* value
TNM stage	Low expression	High expression	
I	13	14	0.134
II	11	6	
III/IV	5	10	

## Data Availability

The data that support the findings of this study are available from the corresponding author upon reasonable request.

## Supplementary Materials

Supplementary Materials Supplementary Figure 1: the expression of four hub genes between cancer group and normal group: A: CCNE2; B: CCNB1; C: MCM7; D: PCNA. Supplementary Figure 2: the expression of four hub genes among the different TNM stages in the cancer group: A: CCNE2; B: CCNB1; C: MCM7; D: PCNA. Supplementary Table: the primers of the four hub genes.
