# Supplementary material for: Identification of Hub Genes as Biomarkers Correlated with the Proliferation and Prognosis in Lung Cancer: A Weighted Gene Co-Expression Network Analysis
Source: Biomed Res Int. 2020 Jun 10;2020:3416807. doi: 10.1155/2020/3416807 (PMC7305540; doi:10.1155/2020/3416807)

| GENE | primer |
| --- | --- |
| CCNB1_F | AATAAGGCGAAGATCAACATGGC |
| CCNB1_R | TTTGTTACCAATGTCCCCAAGAG |
| CCNE2_F | TCAAGACGAAGTAGCCGTTTAC |
| CCNE2_R | TGACATCCTGGGTAGTTTTCCTC |
| MCM7_F | CCTACCAGCCGATCCAGTCT |
| MCM7_R | CCTCCTGAGCGGTTGGTTT |
| PCNA_F | CCTGCTGGGATATTAGCTCCA |
| PCNA_R | CAGCGGTAGGTGTCGAAGC |
|  |  |

Supplementary table: primers of the four hub-genes

supplementary figure 1: The expression of four hub-gene between cancer group and normal group


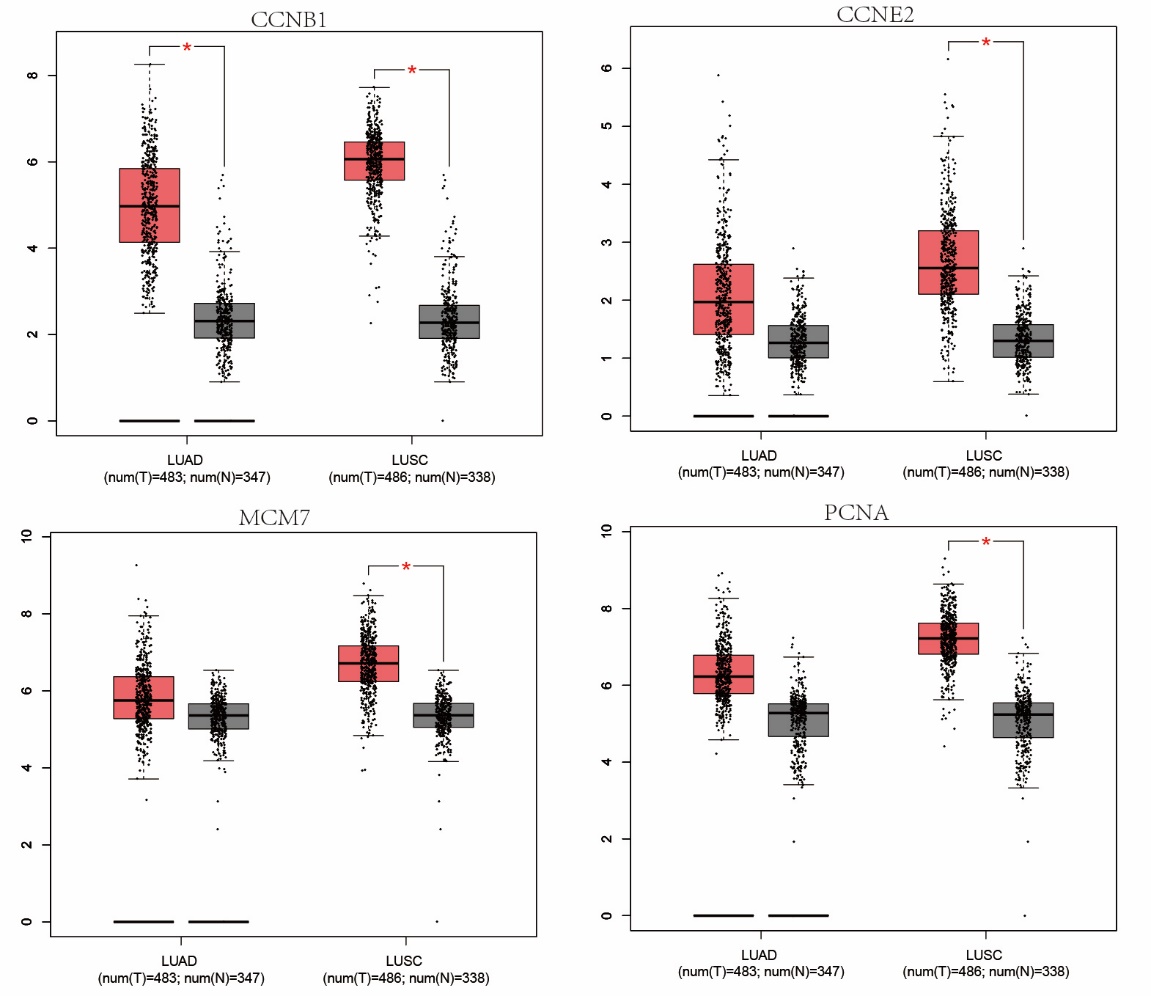


supplementary figure 2: The expression of four hub-gene among the different TNM stage in cancer group


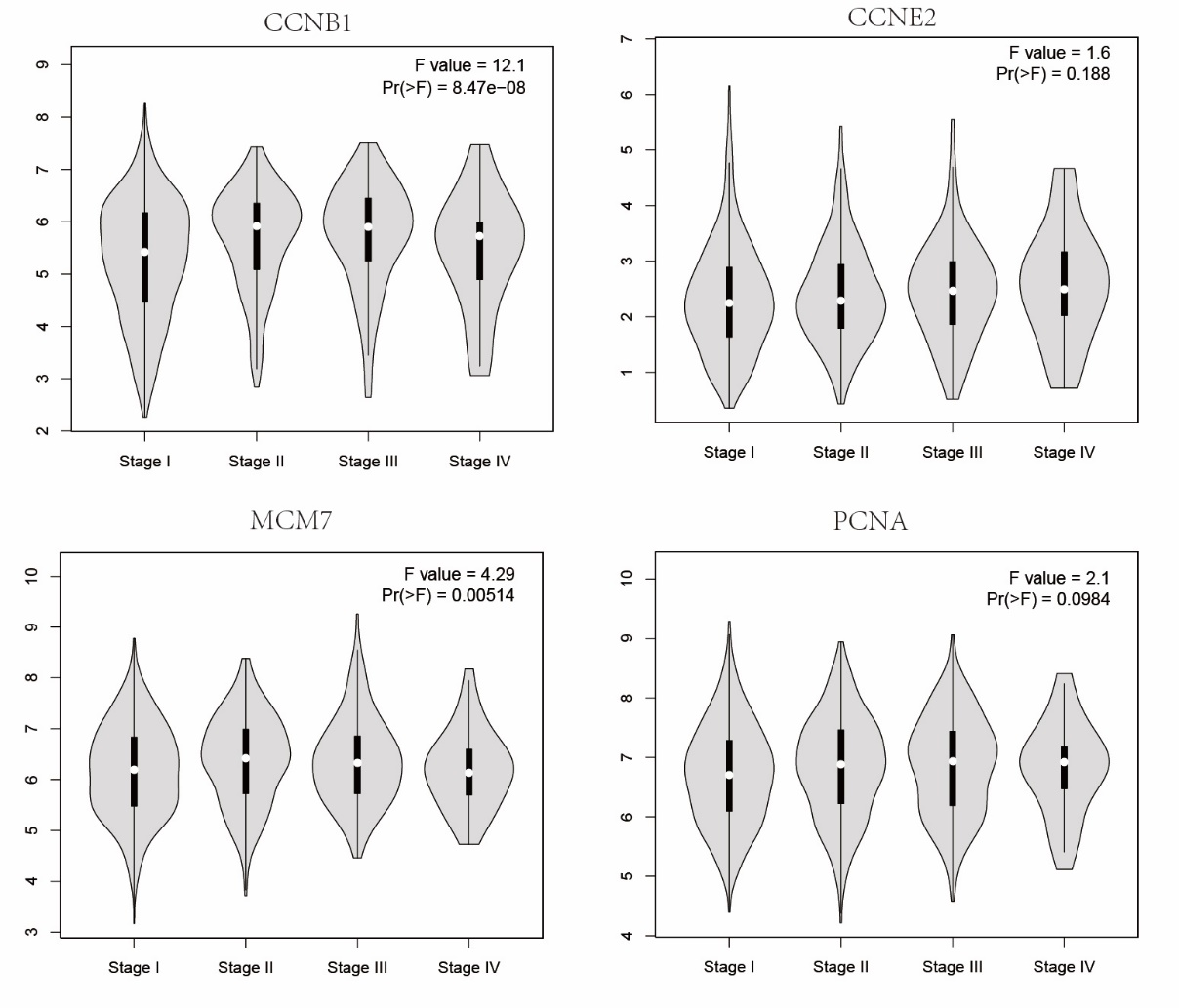

Supplement: Supplementary Materials — Supplementary Figure 1: the expression of four hub genes between cancer group and normal group: A: CCNE2; B: CCNB1; C: MCM7; D: PCNA. Supplementary Figure 2: the expression of four hub genes among the different TNM stages in the cancer group: A: CCNE2; B: CCNB1; C: MCM7; D: PCNA. Supplementary Table: the primers of the four hub genes. [file 3416807.f1.docx]
